# Interocular symmetry and asymmetry in ocular health and disease

**DOI:** 10.3389/fmed.2025.1626329

**Published:** 2025-09-23

**Authors:** Hongli Cui, Zhengwei Zhang

**Affiliations:** ^1^Department of Ophthalmology, Wuxi No. 2 People’s Hospital, Affiliated Wuxi Clinical College of Nantong University, Wuxi, Jiangsu, China; ^2^Department of Ophthalmology, Jiangnan University Medical Centre, Wuxi, Jiangsu, China

**Keywords:** interocular symmetry, interocular asymmetry, ocular health, ocular diseases, early-stage disease diagnosis, optical coherence tomography, optical coherence tomography angiography

## Abstract

In various ocular tissues, the presence of symmetry and asymmetry not only influences physiological functions but also demonstrates significant correlations with the pathogenesis and progression of multiple ophthalmic disorders. Under healthy conditions, ocular structures typically maintain a high degree of bilateral symmetry, ensuring stability and efficacy in visual perception. However, subtle interocular asymmetries may emerge due to factors including aging and environmental exposures, providing critical biological insights into visual functionality and ocular health maintenance. Under pathological circumstances, specific structural asymmetries often serve as early indicators of disease progression. Quantitative analysis of multilayer structural alterations using advanced ophthalmic imaging modalities offers valuable reference data for early disease detection and therapeutic interventions. A comprehensive investigation of ocular symmetry and asymmetry facilitates precise characterization of normative ocular architecture, thereby establishing a theoretical foundation for elucidating disease mechanisms and developing early diagnostic strategies. This multidimensional approach enhances our understanding of ocular pathophysiology and informs evidence-based clinical decision-making.

## 1 Introduction

The symmetry and asymmetry of binocular ocular structures not only directly influence the optimization of visual function but also hold significant implications for overall ocular health (1, 2). In healthy populations, adult ocular structures, including the cornea, ganglion cell – inner plexiform layer (GC-IPL), foveal avascular zone (FAZ), and photoreceptor layer, demonstrate remarkable interocular symmetry, which ensures the stability and efficacy of visual perception. However, subtle interocular variations arising from genetic predisposition, environmental influences, and physiological variations may indicate pathological conditions. For instance, early diagnosis of keratoconus (KC) relies on the quantitative analysis of corneal parameter asymmetry. At the same time, measurable disparities in retinal ganglion cell complex (GCC) thickness emerge during the initial stages of glaucoma. Notably, systemic disorders such as multiple sclerosis and cognitive frailty (CF) may manifest detectable ocular asymmetries that reflect underlying systemic pathologies. Research on interocular asymmetries not only facilitates the diagnosis of ocular pathologies but also enables a comprehensive assessment of systemic disorders through ocular manifestations. Advanced ocular imaging modalities, particularly optical coherence tomography (OCT) and optical coherence tomography angiography (OCTA), have revolutionized the detection and quantification of these asymmetries, offering critical data for early clinical intervention (3). Nevertheless, standardized reference parameters for interocular symmetry across diverse demographic populations and age groups remain undefined. In contrast, pathological associations between ophthalmic and systemic diseases and ocular symmetry/asymmetry profiles remain incompletely elucidated, necessitating comprehensive investigation. This review synthesizes current literature to systematically examine the spectrum of interocular variations in both physiological and pathological states while analyzing their underlying mechanisms. The findings aim to establish theoretical foundations for early disease detection and intervention strategies, thereby advancing ophthalmological practice from traditional “single-eye-centric” diagnostic approaches toward a comprehensive “binocular systemic evaluation” paradigm (1).

## 2 Interocular symmetry and asymmetry in ocular physiology

### 2.1 Cornea

The cornea—a transparent, slightly prolate ellipsoid structure located at the anterior ocular surface—serves as a critical component of the refractive system. Accumulating evidence indicates high interocular symmetry in corneal biometric parameters, including keratometry, and in central corneal thickness (CCT), elevation maps of the anterior and posterior cornea, and corneal endothelial cell density (CECD) in healthy individuals (4–6). This inherent physiological symmetry constitutes the anatomical foundation of the binocular system medicine paradigm, which posits that healthy corneas maintain a precise morphological equilibrium. This symmetry exhibits no significant correlation with age, sex, refractive errors, or systemic metabolic parameters (e.g., blood pressure and oxygen saturation). However, myopia may mildly attenuate symmetry, while the coefficient of variation in endothelial cell size demonstrates a weak inverse association with aging, suggesting potential age-related declines in bilateral corneal endothelial stability (6, 7). Recent longitudinal studies reveal progressive age-dependent trends toward corneal parameter asymmetry. Kelekele et al. established population-specific thresholds for healthy Congolese cohorts, identifying absolute interocular differences exceeding 15.4 μm for CCT and 182 cells/mm^2^ for CECD as clinically significant outliers, warranting etiological evaluation (Table 1) (6). These thresholds hold diagnostic relevance for refractive surgery candidate screening, post-keratoplasty rejection risk stratification, monitoring of long-term contact lens users, and early detection of KC (8). Integration of bilateral asymmetry mapping into corneal imaging systems could enhance preoperative assessments and routine ophthalmic evaluations. By leveraging the symmetry of healthy corneas as a reference standard, the binocular system medicine paradigm has been refined. Notably, the inherent binocular symmetry of corneal architecture has inspired therapeutic innovations for unilateral corneal pathologies. Biosynthetic corneal substitutes, designed through mirroring parameters from the healthy contralateral cornea, demonstrate promise in minimizing postoperative refractive errors and improving visual outcomes (9, 10).

**Table 1:** Normative/pathological asymmetry thresholds for ocular structures and pathological states.

Structure/pathology	Parameter	Normative/pathological asymmetry thresholds	Clinical significance	Reference
Cornea	CCT	≤15.4 μm	Exceeding values suggest pathology, applicable for: refractive surgery indications, graft rejection risks, contact lens surveillance, and keratoconus screening.	(6)
	CECD	≤182 cells/mm^2^		
Retina	macular regions (preterm infants)	16.3 ± 16.6 μm	Values exceeding this threshold may indicate pathological alterations during child development.	(15)
	RNFL	≤9 μm	Exceeding this value suggests abnormal findings, potentially indicating early structural retinal damage.	(28)
	BMO-MRW	≤49 μm		
KC	Max-K	0.96 D	Exceeding measurements may signal very early-stage keratoconus.	(55)
	maximum posterior elevation	10.76 μm		
	maximum anterior elevation	4.95 μm		
	IAI	0.494	Values above this threshold can differentiate normal eyes from KCS.	(56)
		0.778	Exceeding this value helps distinguish normal eyes from KC.	
Glaucoma	GCC	8 μm	Measurements surpassing this level may indicate early-stage glaucoma.	(63)
		20 μm	Measurements surpassing this level may indicate late-stage glaucoma.	
CCF	IOP	2.92 ± 2.29 mm Hg	Values exceeding this parameter may suggest possible normal-tension CCF.	(88)
CF	GC-IPL	17 μm	Measurements above this threshold could indicate potential CF.	(89)

CCT: central corneal thickness; CECD: corneal endothelial cell density; RNFL: retinal nerve fiber layer; BMO-MRW: Bruch’s membrane opening minimum rim width; KC: keratoconus; KCS: keratoconus suspect; Max-K: maximum keratometry; IAI: Interocular Asymmetry Index; GCC: ganglion cell complex; CCF: carotid-cavernous fistulas; IOP: intraocular pressure; CF: cognitive frailty; GC-IPL: ganglion cell-inner plexiform layer.

### 2.2 Retina

The structural and functional symmetry of retinal laminar architecture plays a crucial regulatory role in optimizing retinal information processing efficiency, with interocular symmetry minimizing errors in visual signal transduction and processing (11, 12). Developmental studies of macular characteristics in children and adolescents reveal robust binocular symmetry in macular thickness profiles (13). Quantitative benchmarks include central foveal thickness <22 μm, extrafoveal regions <40 μm, and peripapillary retinal nerve fiber layer (RNFL) thickness averaging 16–17 μm, with symmetrical cup-to-disc ratios (C/D) (14). A longitudinal investigation evaluating macular thickness variations and interocular symmetry in preterm children aged 5–8 years demonstrated that premature birth disrupts normative macular development, inducing structural anomalies. Notably, high bilateral symmetry persisted across most macular regions (mean interocular difference: 16.3 ± 16.6 μm), with significant disparities localized to the central fovea (Table 1) (15). These findings suggest that prematurity-associated retinal abnormalities predominantly manifest as central thickening and morphological irregularities rather than binocular asymmetry. This preserved symmetry implies that prematurity itself does not constitute a primary determinant of interocular disparity, providing critical insights for clinical differentiation of developmental retinal pathologies in preterm populations (15).

The binocular symmetry of retinal layer structures provides the theoretical foundation for the binocular system medicine paradigm, enabling the detection of pathologies obscured in monocular assessments. Significant interocular structural asymmetry in the fovea of preterm infants may indicate pathological alterations during macular development. While physiological asymmetry of the RNFL exists within normative population ranges, deviations beyond these parameters suggest potential abnormalities. The GC-IPL and FAZ exhibit marked interocular symmetry; values exceeding established threshold ranges warrant clinical vigilance. Clinicians should transcend beyond the isolated assessment of the affected eye, adopting a holistic, binocular system medicine-based evaluation to formulate tailored diagnostic and therapeutic strategies.

#### 2.2.1 Retinal nerve fiber layer

Previous studies demonstrate that the mean interocular difference in global RNFL thickness ranges between −0.9 and 3.58 μm, with distinct spatial distribution patterns (16–21). Except for the superior RNFL sectors (particularly the superonasal sector), right eyes generally exhibit greater thickness than left eyes, with pronounced asymmetry in the superotemporal sector. The interocular RNFL thickness asymmetry profile resembles a “W” shape (22–26).

A study investigating the RNFL thickness distribution in 1,288 healthy young adults demonstrated significant interocular variations (19). The right eye exhibited greater thickness in the global (100.5 μm vs. 100.3 μm in the left eye), temporal (73.1 μm vs. 68.9 μm), and superotemporal (140.6 μm vs. 136.3 μm) sectors. Conversely, the left eye showed thicker RNFL in the superonasal (115.1 μm vs. 104.9 μm) and inferonasal (111.5 μm vs. 109.8 μm) sectors. No statistically significant differences were observed in the nasal (79.7 μm vs. 79.1 μm) or inferotemporal (143.2 μm vs. 143.6 μm) sectors. These findings highlight distinct anatomical and functional asymmetries in RNFL distribution between eyes, emphasizing the need for eye-specific normative databases in clinical assessments of optic nerve health. The prevalence of the percentage of outside normal limits and borderline (BL) RNFL classifications was generally higher than the expected rates of 1 and 4%, respectively, in the temporal sectors and lower than expected in the nasal sectors. The prevalence of global BL classifications was lower than expected (right eye, 2.3%; left eye, 2.6%) (19).

According to ocular dominance and laterality, the physiological thickening of RNFL in visually dominant regions, with right-eye dominance, is observed in approximately 67% of the population (70% male, 65% female). This phenomenon may relate to region-specific rates of retinal ganglion cell attrition (22, 27). Longer axial length (AL) was associated with thinner RNFL globally, nasally, inferotemporally, superotemporally, superonasally, and inferonasally, as well as thicker RNFL temporally (19). Zangalli et al. identified a positive correlation between RNFL asymmetry and Bruch’s membrane opening minimum rim width (BMO-MRW) asymmetry in healthy Brazilian cohorts, while BMO-MRW asymmetry inversely correlated with Bruch’s membrane opening area asymmetry. Neither BMO-MRW nor RNFL asymmetry showed associations with AL (28). The authors proposed thresholds of >49 μm for BMO-MRW and >9 μm for global RNFL interocular differences as indicators of statistically significant asymmetry, potentially suggestive of early structural compromise (Table 1) (28). Vascular analyses by Ly et al. revealed positive correlations between vascular diameter and RNFL thickness, though venous diameter exhibited greater interocular variability in individuals with marked superonasal asymmetry (29). Contrarily, Quach et al. concluded that retinal blood vessel (RBV) thickness and position do not directly drive RNFL asymmetry (26).

A study of 310 healthy children and adolescents (5–17 years) identified right-eye RNFL thinning in superior quadrants but thickening in the nasal and temporal sectors, with interocular differences ranging from −9.0 to 11.0 μm (30). Increasing AL and spherical equivalent (SE) positively correlated with RNFL asymmetry, while sex and age showed no significant associations (30). However, conflicting evidence suggests more pronounced interocular differences in individuals >10 years (31).

#### 2.2.2 Ganglion cell-inner plexiform layer

GC-IPL, a critical retinal structure comprising the ganglion cell layer and inner plexiform layer, serves as a principal component of the GCC. GC-IPL thickness alterations reflect global GCC integrity and hold significant diagnostic value for early detection of glaucoma, optic neuropathies, and neurological disorders (32, 33). In healthy adults, GC-IPL exhibits robust interocular symmetry, with a mean interocular difference of 0.10 ± 2.31 μm. The 2.5th and 97.5th percentiles of the interocular difference were −4.10 μm and +5.00 μm, respectively. This disparity demonstrates an independent positive correlation between interocular AL and SE differences but lacks significant associations with age or sex (33, 34). Conversely, pediatric and adolescent populations display greater GC-IPL asymmetry, with right eyes showing thinner mean, superior, and superonasal GC-IPL measurements (range: −4.0 to 4.0 μm) compared to left eyes (30).

The high interocular symmetry of GCC thickness in healthy individuals underscores its potential as a diagnostic biomarker for differentiating physiological variation from pathological states, particularly in glaucoma screening. Future studies should prioritize establishing population-specific reference values and elucidating longitudinal relationships between GCC asymmetry and disease progression across diverse cohorts.

#### 2.2.3 Foveal avascular zone

**Figure 1:**
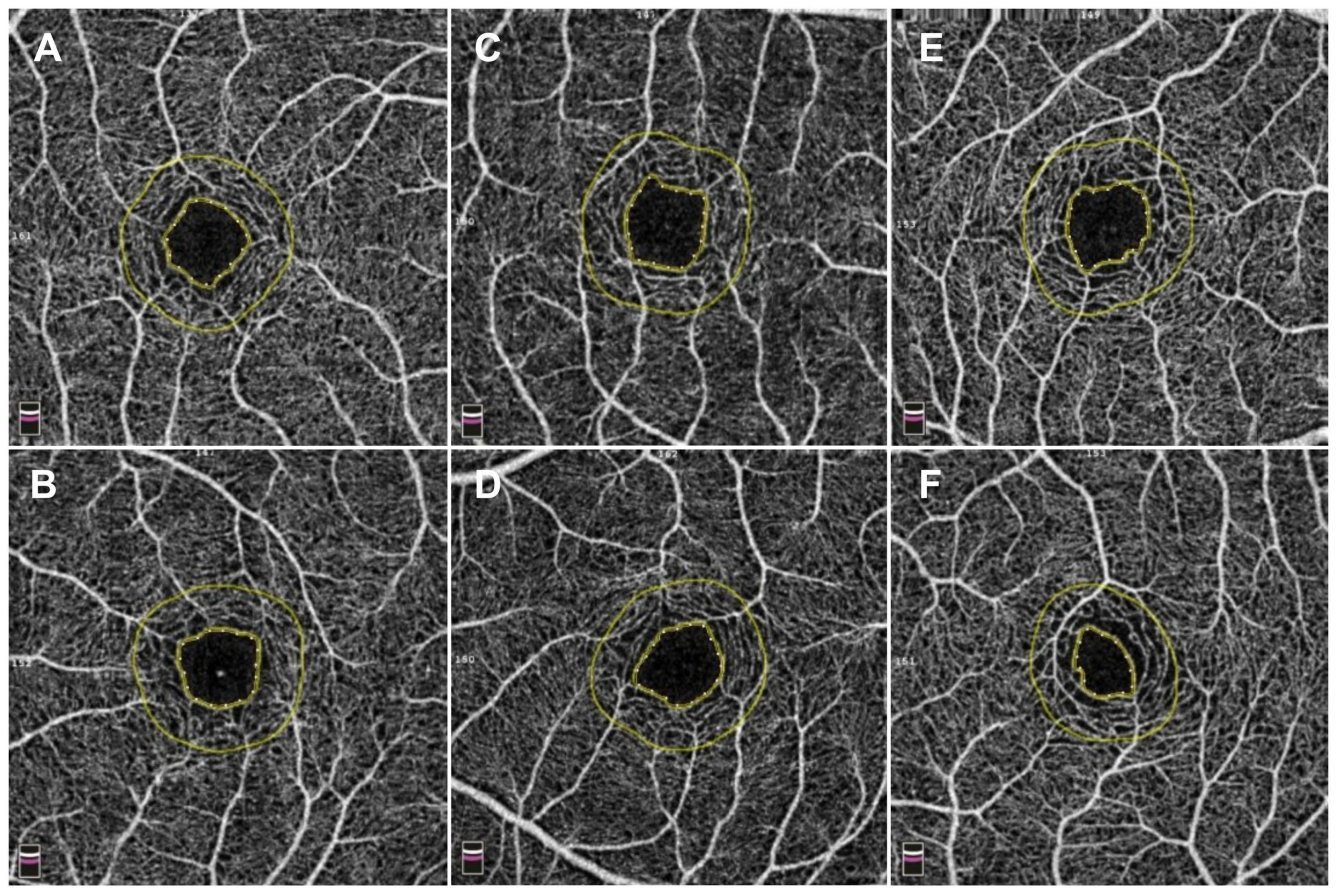
Comparison of three kinds of interocular symmetry of the foveal avascular zone (FAZ). **(A,B)** A 12-year-old adolescent exhibited bilateral FAZ imaging via optical coherence tomography angiography (OCTA) 3 mm × 3 mm scans, with the right eye (quadrilateral configurations) demonstrating a spherical equivalent (SE) of −1.63 D, an axial length (AL) of 24.05 mm, a FAZ area (FAZA) of 0.22 mm^2^, and a perimeter of 1.83 mm, while the left eye (quadrilateral configurations) showed an SE of −1.63 D, an AL of 24.06 mm, a FAZA of 0.23 mm^2^, and a perimeter of 1.83 mm. With no clinically significant interocular differences in SE or AL, the FAZ morphology demonstrated marked symmetry. **(C,D)** A 10-year-old adolescent exhibited bilateral FAZ imaging via OCTA 3 mm × 3 mm scans, with the right eye (quadrilateral configuration) demonstrating an SE of −1.75 D, an AL of 24.95 mm, a FAZA of 0.26 mm^2^, and a perimeter of 2.01 mm, while the left eye (pentagonal configuration) showed an SE of −1.75 D, an AL of 24.97 mm, a FAZA of 0.24 mm^2^, and a perimeter of 1.89 mm. Despite negligible interocular SE and AL variance, FAZ morphology displayed moderate symmetry. **(E,F)** A 10-year-old adolescent exhibited bilateral FAZ imaging via OCTA 3 mm × 3 mm scans, with the right eye (quadrilateral configuration) demonstrating an SE of −1.50 D, an AL of 24.24 mm, a FAZA of 0.25 mm^2^, and a perimeter of 2.01 mm, while the left eye (irregular configuration) showed an SE of −1.75 D, an AL of 24.43 mm, a FAZA of 0.13 mm^2^, and a perimeter of 1.47 mm. With clinically insignificant SE and AL interocular differences, FAZ morphology exhibited marked asymmetry.

The foveal avascular zone (FAZ), the retinal region of the highest visual acuity, serves as an indirect biomarker of macular microcirculatory dynamics. Previous studies have established robust interocular symmetry in the FAZ area (FAZA) (35, 36). Qiang et al. expanded these findings, demonstrating bilateral symmetry not only in FAZA but also in foveal density-300 (vascular density in a 300-μm wide annulus surrounding the FAZ), which exhibits a significant negative correlation with central retinal thickness (37). Notably, right eyes demonstrate higher vascular density in the superficial retinal capillary plexus (SCP), deep retinal capillary plexus (DCP), and choriocapillaris compared to left eyes—a disparity postulated to correlate with ocular dominance (25, 38). Longitudinal analyses reveal age-dependent increases in FAZA, with interocular differences amplifying over time. Annual FAZA expansion rates average 0.0014 mm^2^ (0.63%) for SCP and 0.0011 mm^2^ (0.20%) for DCP in healthy adults (38, 39). These insights provide novel perspectives for managing retinal disorders characterized by marked interocular FAZ disparities. Quantitative assessment of FAZA differences between affected and contralateral eyes may enhance precision in monitoring disease progression and therapeutic efficacy (40). The FAZ exhibits high interocular symmetry in quantitative metrics. However, our unpublished data reveal that FAZ morphology is not entirely symmetrical. After accounting for the confounding effects of spherical equivalent and AL, a substantial proportion of FAZ still demonstrate morphological asymmetry. This finding indicates that numerical symmetry does not necessarily reflect morphological symmetry (Figure 1). Additionally, it should be noted that the morphological symmetry of the FAZ between both eyes exhibits considerable variability among healthy individuals, which could be beneficial for improving binocular system medicine.

### 2.3 Choroid

**Figure 2:**
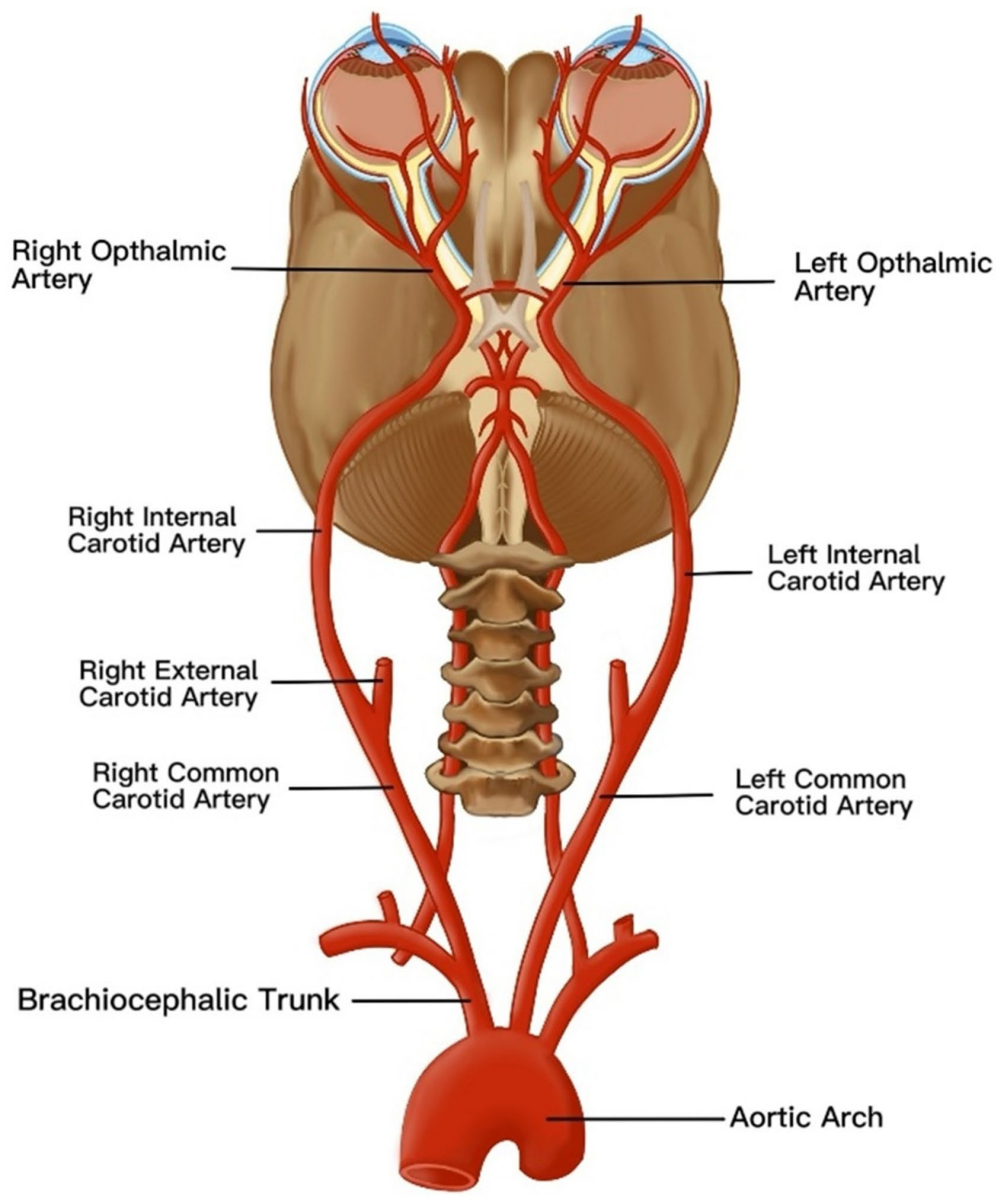
An anatomical map of vascular anatomy affecting choroidal thickness asymmetry. The ophthalmic artery, a branch of the internal carotid artery, originates from the common carotid artery (CCA). Notably, the right CCA arises from the brachiocephalic trunk in the cervical region, whereas the left CCA originates directly from the aortic arch in the thoracic cavity. This inherent asymmetry in vascular architecture induces divergent hemodynamic profiles in the ophthalmic arteries, resulting in differential choroidal perfusion. Consequently, these hemodynamic disparities manifest as increased choroidal thickness in the right eye compared to the left eye.

Situated between the retina and sclera, the choroid supplies blood and nutrients to the retinal pigment epithelium (RPE) and outer retinal layers (41). Its functional status can be quantitatively assessed through the choroidal vascularity index (CVI) and choroidal thickness (ChT), both of which are critical parameters for evaluating posterior segment pathologies (42). Kim et al. conducted OCT-based quantitative analyses of ChT symmetry in healthy young adults, revealing bilateral symmetry across all choroidal regions (43). However, the nasal peripapillary and peripheral areas had relatively low correlation coefficients compared to the macular areas. In addition, the bilateral CT differences were 32.60 ± 25.80 μm in the macular area, 40.67 ± 30.58 μm in the nasal peripapillary area, and 56.03 ± 45.73 μm in the peripheral area. Most studies consistently report greater ChT in the right eyes compared to the left eyes (42, 44–46). Proposed mechanisms for this asymmetry include the following: (1) anatomic vascular asymmetry: The right common carotid artery (CCA) originates from the brachiocephalic trunk in the neck, whereas the left CCA arises directly from the aortic arch in the thorax. As the ophthalmic artery branches from the internal carotid artery to perfuse the choroidal vasculature (Figure 2), the shorter anatomic course and reduced vascular resistance in the right carotid system may enhance blood flow velocity and choroidal perfusion, resulting in greater right-eye ChT (47) and (2) Ocular dominance effects: Approximately 67% of the population exhibits right-eye dominance (27). Dominant eyes may regulate local ChT through metabolic demand modulation, though this hypothesis requires further validation (48).

Current evidence suggests no significant associations between age, sex, or intraocular pressure (IOP) with interocular ChT disparities (42, 43). However, AL emerges as a critical determinant of nasal and foveal ChT asymmetry, with eyes exceeding 23.50 mm AL demonstrating amplified interocular ChT differences (43, 49). Future investigations should prioritize elucidating additional modulators of topographic ChT distribution and interocular variability in healthy populations. Such efforts will enable individualized physiological thresholds for intraocular variation and binocular disparity, facilitating early identification of pathological choroidal alterations (49). Therefore, choroidal assessment under the binocular system medicine paradigm facilitates the discrimination between physiological asymmetry and pathological deviation, offering potential clinical value for early diagnosis and management of related ocular diseases.

### 2.4 Others

The photoreceptor layer, comprising rod and cone photoreceptors, constitutes the light-sensitive neural retina. Topographic analyses of cone photoreceptors in the macular region reveal that, while interindividual density variations are substantial, total cone counts remain remarkably consistent between eyes in healthy populations. Bilateral symmetry has been observed in cone density, mosaic regularity, and area encompassing the most densely packed cells in subjects with normal vision (48, 49). Similarly, rod photoreceptors demonstrate high interocular concordance in distribution patterns and density profiles (50). These physiological symmetry characteristics provide critical insights into the normative macular architecture and offer potential biomarkers for the early detection of photoreceptor-related pathologies. Furthermore, such symmetry metrics hold significant translational value for guiding experimental therapeutic design and optimizing clinical trial protocols through anatomically matched interocular comparisons (51).

The optic disc, an anatomical blind spot devoid of photoreceptors and pigmented epithelium, demonstrates notable interocular symmetry in pediatric populations. Studies reveal strong bilateral correlations in foveal minimum thickness and C/D, with C/D symmetry being particularly pronounced: 95% of children exhibit interocular C/D values <0.25 and minimal mean differences. Quantitative analyses reveal interocular measurement disparities of approximately 0.8 mm^2^ for optic disk and neural rim areas and 0.5 mm^2^ for optic cup area (13). Age and sex demonstrate no significant associations with interocular optic disc asymmetry, whereas anisometropia positively correlates with interocular optic disc area disparities (30).

The RBV distribution analysis revealed the mean (standard deviation) number of RBV measured per eye was 15.0. The position of the superotemporal vein and artery was superior in the left eyes than in the right eyes, by 2.4 and 3.7 degrees, respectively (26). No statistically significant interocular differences were observed in vascular diameter (26, 52). However, right eye venous diameter demonstrated greater dimensions than the left eye in populations exhibiting significant superonasal quadrant RNFL thickness asymmetry, with observed RNFL thickening demonstrating a positive association with increased vascular (particularly arterial) diameter. This phenomenon may be partially attributed to spatial correspondence within anatomical configurations (26). Notably, RBV diameter accounted for 24% of local RNFL thickness variability, a substantially higher explanatory power than previous investigations based on global and quadrant-specific RNFL thickness parameters combined with demographic (age) and ocular characteristics (AL, refractive error, IOP, C/D, optic disc, and rim area) (29).

The pronounced symmetry observed in the photoreceptor layer and pediatric optic disc parameters (particularly the C/D) provides critical normative reference values for the binocular system medicine paradigm. Concurrently, investigations of the retinal vasculature reveal the complexity inherent in binocular system medicine: while inherent heterogeneity exists in vascular topography between eyes, vessel diameter exhibits no significant interocular difference. Binocular comparative analysis uncovers pathophysiological associations undetectable by monocular assessment—specifically, a robust correlation between superonasal RNFL thickness asymmetry and ipsilateral vascular diameter alterations. Notably, RBV diameter demonstrated substantially greater explanatory power for localized RNFL variation than traditional parameters. These findings advance the binocular system medicine paradigm, providing a novel systems biology perspective for understanding physiological and pathological changes within the retinal microenvironment.

## 3 Ocular symmetry and asymmetry in pathological states

### 3.1 Keratoconus

Interocular corneal asymmetry may serve as a potential early warning indicator for various corneal pathologies, including KC. Early-stage corneal diseases often manifest as alterations in bilateral corneal symmetry, underscoring the critical role of interocular corneal parameter measurement in early diagnosis, therapeutic efficacy evaluation, and prognostic management of corneal disorders (9). KC serves as a paradigmatic example of pathological system imbalance within the binocular system medicine framework.

KC—a bilateral yet asymmetric non-inflammatory corneal ectasia—is characterized by progressive corneal thinning and protrusion, leading to increasing myopia, irregular astigmatism, and visual impairment (53). KC exhibits significant interocular asymmetry in corneal keratometry, SE, posterior corneal elevation, and corneal thickness, with moderate symmetry in CCT. Such disparities amplify with disease progression, and abnormal changes in the worst-affected eye can be detected at subclinical stages (54–56). Recent diagnostic paradigms for KC have expanded from unilateral evaluation to incorporate interocular asymmetry analysis. Henriquez et al. quantified interocular differences in very early keratoconus (VEKC), reporting mean bilateral disparities of 0.96 D in maximum keratometry (Max-K), 10.76 μm in maximum posterior elevation, and 4.95 μm in maximum anterior elevation—values significantly exceeding those observed in high ametropia and normal corneas (Table 1) (55). Building on this, Arnalich et al. demonstrated a positive correlation between elevated interocular asymmetry in KC patients and progression risk, proposing Max-K asymmetry as a biomarker for progression monitoring. Younger patients with marked Max-K asymmetry require close surveillance to mitigate disease exacerbation (57).

In the diagnosis of keratoconus, traditional approaches rely on monocular parameters; however, during the keratoconus suspect (KCS) stage, corneal morphological changes are subtle and often overlap with normal corneas, leading to underdiagnosis. Consequently, recent research posits that keratoconus is inherently a bilateral asymmetrically progressive disease, with detectable asymmetry manifesting even in KCS. To quantify this characteristic, this study introduces a novel diagnostic metric: the Interocular Asymmetry Index (IAI), which is designed to enhance the diagnosis of both KC and KCS by quantifying symmetry differences in bilateral corneal parameters (56). This index integrates interocular differences in posterior corneal elevation, thinnest corneal thickness, and Max-K into a composite score ranging from 0 to 1, where higher values indicate greater asymmetry (56). An IAI threshold exceeding 0.494 distinguishes normal from KCS eyes with 67.1% sensitivity and 97.1% specificity, while a threshold above 0.778 differentiates normal from KC eyes with 95.0% sensitivity and 96.2% specificity (Table 1). By quantifying the temporal evolution of interocular corneal morphological disparities, IAI provides a particular tool for early keratoconus detection. This metric can be integrated into clinical screening protocols, serving as a critical adjunct for preoperative risk assessment in refractive surgery and monitoring disease progression. The IAI, a pivotal quantitative diagnostic index in binocular system medicine, systematically consolidates interocular differences across multiple key parameters. This methodology overcomes limitations inherent in the monocular assessment for threshold cases, significantly enhancing the sensitivity and specificity for the early detection of KC-associated system imbalance (56).

Current screening protocols recommend combining interocular asymmetry analysis with unilateral assessment for KC detection. Patients presenting with significant Max-K asymmetry or elevated IAI at initial evaluation warrant intensified longitudinal monitoring to identify subclinical KC and implement timely interventions, thereby minimizing progression risk (56–58).

### 3.2 Glaucoma

Glaucoma is an optic neuropathy characterized by progressive degeneration of retinal ganglion cells and their axons, accompanied by a pathognomonic visual field defect (VFD) (34). Binocular system medicine conceptualizes glaucoma as a disease affecting the entire neurosensory visual system, with early manifestations frequently presenting as an interocular system imbalance. Studies demonstrate elevated interocular asymmetry in peripapillary and macular vessel density among glaucoma patients. When such asymmetry manifests in early-stage disease, it suggests that vascular density reduction may precede detectable structural damage (59). Subtype-specific analyses of normal-tension glaucoma (NTG) reveal distinct patterns: in myopic NTG, eyes with higher refractive error exhibit more severe VFD, whereas in non-myopic NTG, eyes with elevated IOP exhibit faster progression among patients (60). Age-related progression correlates with heightened interocular asymmetry, driving vertical C/D enlargement, RNFL thinning, and aggravated VFD, with functional deterioration more pronounced in non-dominant eyes (60). Amplitude change (Ac) is the absolute reduction in pupil diameter under photic stimulation, and Ac percentage represents Ac normalized to baseline pupil diameter, reflecting relative constriction intensity. Interocular Ac percentage disparity emerges as the strongest predictor of mean deviation asymmetry, RNFL, and GCC thickness differences. This parameter demonstrates superior predictive capacity in angle-closure glaucoma compared to open-angle subtypes, likely due to the inherently asymmetric damage pattern in angle-closure disease (61). The role of myopia as a progression risk factor remains contentious (62).

The GCC exhibits high interocular symmetry in healthy populations but significantly increased thickness asymmetry in glaucoma: a significantly faster GC-IPL thinning in the pseudoexfoliation glaucoma when compared with the other two types (−0.31 μm/y normal eyes, −0.49 μm/y open-angle glaucoma, −1.46 μm/y pseudoexfoliation glaucoma) (63). In early glaucoma, GCC analysis (particularly inferotemporal quadrant thickness) surpasses peripapillary RNFL in sensitivity. Interocular GCC thickness differences >8 μm in superior and inferior macular quadrants serve as early disease markers. Moderate-stage progression correlates strongly with inferior RNFL thinning, while late-stage differences >20 μm require concurrent visual field confirmation (Table 1) (63). These findings underscore binocular system medicine’s diagnostic superiority in glaucoma: quantification of GCC thickness asymmetry sensitively detects early, localized neurosensory system imbalance, with particular diagnostic value when monocular parameters remain within normal limits.

Glaucomatous VFD arises from impaired retinal ganglion cell signaling. While progression speed shows no association with CCT, it positively correlates with interocular corneal hysteresis (CH) disparity: each 1 mm Hg increase in CH asymmetry elevates progression rate differences by 34% (Table 1) (62, 64). This reveals another critical dimension of binocular system medicine that system imbalance extends beyond the retina to ocular biomechanical properties, where interocular CH disparity emerges as a significant predictor of differential binocular visual field progression. Two novel indices refine progression monitoring in primary open-angle glaucoma (POAG): intereye mean deviation asymmetry index (iMAI) and intereye hemifield visual sensitivity asymmetric index (ihVAI). Unilateral VFD eyes exhibit significantly higher progression rates (−1.27 dB/year; iMAI = 7.93 ± 1.93 dB) and ihVAI = 12.93 ± 4.49 dB vs. bilateral VFD eyes (−0.64 dB/year; iMAI 2.62 ± 1.57 dB; ihVAI 5.84 ± 3.46 dB) or bilateral opposite visual field hemifield (−0.32 dB/year; iMAI 2.50 ± 1.69 dB; ihVAI 11.46 ± 4.79 dB) (65).

Stage-dependent variations in structure–function relationships necessitate multimodal asymmetry assessment. Parameters, including Ac, CH, and GCC analysis, collectively enhance detection sensitivity (63). The integrated application of these binocular asymmetry metrics constitutes the cornerstone of binocular system medicine-based glaucoma assessment. Clinicians should prioritize interocular differential treatment strategies to optimize personalized interventions, particularly in patients demonstrating marked asymmetry indices (64).

### 3.3 Refractive error

**Figure 3:**
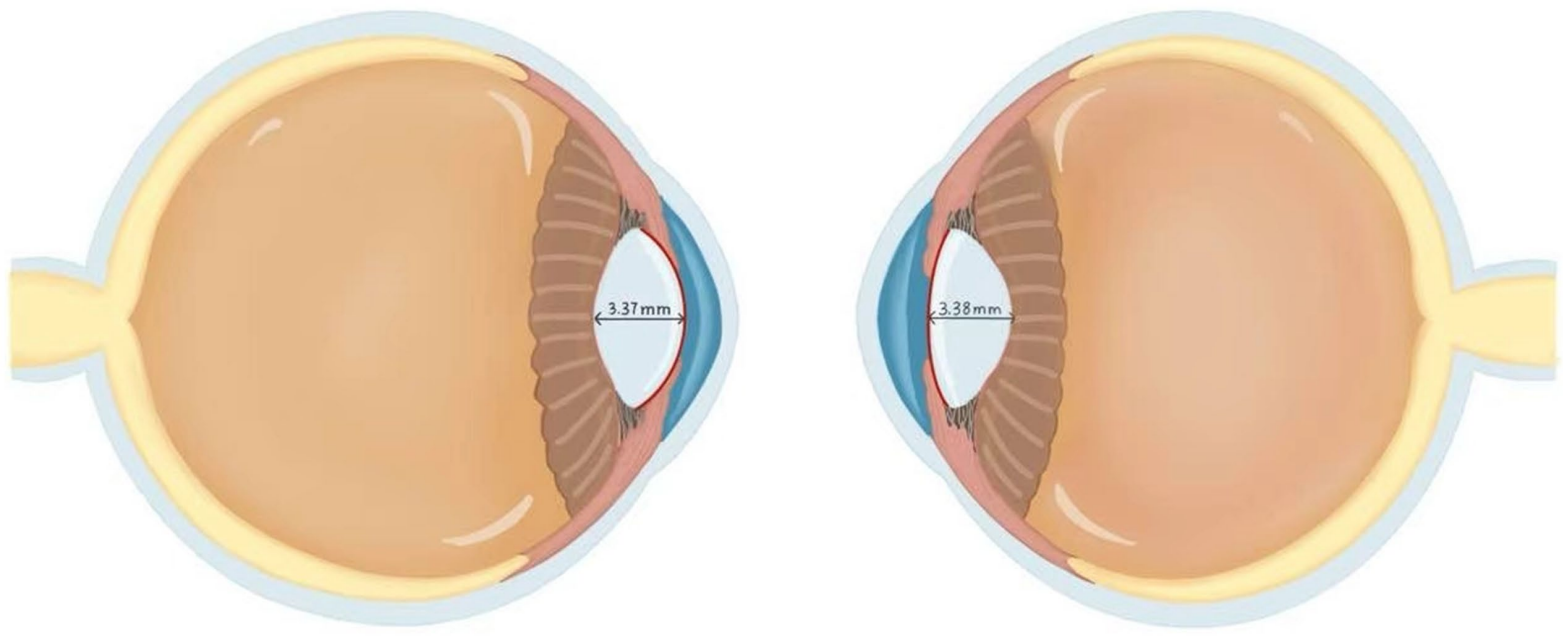
Comparison of the lens between high myopia and normal eyes. The left panel demonstrates a highly myopic eye, while the right panel shows the contralateral normal eye. Both eyes demonstrated equivalent central lens thickness measurements of 3.37 mm. In the high myopia group, the lens radius of curvature measured 13.07 ± 1.49 mm, which was significantly lower than the 13.40 ± 1.30 mm observed in the contralateral normal eyes. This finding resolves Brown’s lens paradox theory. Although increased ALR would theoretically enhance refractive power, the lens adapts to axial elongation via self-compression rather than a thinning morphological change that paradoxically reduces total lens power. This morphological distinction suggests that the high myopia group primarily achieves refractive compensation through increased anterior lens surface curvature rather than through alterations in central lens thickness. This figure was created based on data from the reference (66).

Refractive error denotes an ocular condition wherein parallel light rays, under accommodative relaxation, fail to converge onto a precise retinal focal point following refraction through the optical system, resulting in diminished visual acuity. Anisometropia, a subtype of refractive error, fundamentally represents a systemic imbalance within the binocular refractive system (encompassing cornea and lens). Within the binocular system medicine paradigm, anisometropia serves as a pivotal model for understanding the synergistic coordination or breakdown of the binocular refractive system. A cross-sectional study of 91 children and adolescents (aged 6–18 years) with unilateral high myopia investigated interocular differences in crystalline lens morphology and their association with anisometropia. Key findings revealed that high myopic eyes exhibited significantly greater AL (26.21 ± 1.04 mm vs. 23.90 ± 0.87 mm in contralateral eyes), reduced lens power (LP) (3.79 ± 1.59 D vs. 24.61 ± 1.77 D), flatter anterior lens radius of curvature (ALR) (13.07 ± 1.49 mm vs. 13.40 ± 1.30 mm), and steeper posterior ALR (6.05 ± 0.43 mm vs. 5.95 ± 0.40 mm) compared to contralateral controls. Central lens thickness showed no interocular disparity (3.37 ± 0.25 mm vs. 3.38 ± 0.16 mm) (66). A multivariate analysis adjusted for age and sex identified AL and ALR disparities as significant contributors to interocular SE differences. The study posits that the lens in more myopic eyes had lower LP, resembling a compensatory effect for axial elongation. Paradoxically, an increased curved anterior surface theoretically enhances LP, but the LP is reduced in high myopic eyes. The finding that the crystalline lens in highly myopic eyes achieves optical compensation through increased anterior surface curvature (without significant thickness alteration) supports Brown’s lens paradox theory. This study elucidates the paradox by demonstrating that the lens compensates for axial elongation via active adjustment of its internal refractive properties rather than passive zonular traction (Figure 3). These findings elucidate the bilateral lens asymmetry in unilateral high myopia and its mechanistic role in anisometropia (66).

Myopia, a principal subtype of refractive error, is characterized by premature focal convergence of parallel light rays anterior to the retina due to excessive ocular refractive power or axial elongation. A multicenter study of Chinese myopic patients revealed high interocular symmetry in corneal parameters, including the curvature of the anterior and posterior corneal surface, corneal diameter, corneal thickness, corneal volume, corneal eccentricity, and asphericity, with moderate symmetry observed in corneal astigmatism (9). Further analysis of healthy Caucasian populations corroborated these findings while revealing nuanced interocular variations: in some cases, 33% did not exhibit symmetry, with the two eyes displaying different patterns. The two most commonly observed conditions were the two eyes showing with-the-rule and oblique astigmatism (13%), or against-the-rule and oblique astigmatism (12%), respectively. Age and sex did not affect the likelihood of exhibiting corneal astigmatic mirror, whereas the degree of myopia exhibited a positive correlation with interocular discrepancies in corneal astigmatism (7).

In patients with high myopia, posterior staphyloma (PS) is a frequent comorbidity. PS was defined as a localized outpouching of the ocular wall with a radius of curvature less than the surrounding curvature of the ocular wall. A study analyzing the bilaterality and symmetry of PS in 259 high myopia patients (AL ≥ 26 mm) revealed that 92.8% of eyes exhibited PS, with 85.6% of patients presenting bilateral PS (67). Bilateral PS eyes demonstrated significantly greater AL (30.27 ± 2.58 mm vs. 28.45 ± 2.04 mm), higher atrophic component (2.58 ± 0.74 vs. 2.04 ± 0.87), and increased prevalence of severe pathologic myopia (PM) (60.84% vs. 37.5%) compared to unilateral PS eyes. Asymmetric PS eyes exhibited worse best-corrected visual acuity (BCVA), longer AL, more severe atrophic component, and significantly higher prevalence of PM and severe PM. Investigations of PS yield critical insights for binocular system medicine applications in pathological myopia: the high bilaterality of PS in high myopia signifies a systemic pathological process affecting both eyes rather than an isolated monocular event. PS asymmetry serves as an indicator of exacerbated systemic imbalance, signifying advanced disease stages concomitant with more severe functional and structural damage. These findings suggest that PS in high myopia is predominantly bilateral, with symmetry associated with less advanced disease. At the same time, asymmetry likely reflects advanced pathological changes, underscoring the complex interplay of genetic and environmental factors in PS morphology (68).

### 3.4 Amblyopia

Amblyopia is defined as reduced BCVA in one or both eyes that is below the norm for the patient’s age, resulting from abnormal cerebral visual processing during early visual development rather than organic pathology of the eye itself. Anisometropia (SE ≥ 1.00 D between the two eyes) is a major risk factor for amblyopia, accounting for 24 to 37% of all amblyopia cases (69). It is noteworthy that hyperopic anisometropia ≥1.00 D can induce amblyopia, whereas myopic anisometropia typically requires a difference ≥3.00 D to cause amblyopia (69). Deborah et al. tested 30 children with deprivation amblyopia and 59 age-matched controls with normal vision and found that motion-defined form perception deficits were prevalent in 90% of amblyopic eyes and 40% of fellow eyes. Poorer motion-defined form perception in amblyopic eyes was associated with poorer visual acuity, poorer binocular function, greater interocular suppression, and the presence of nystagmus. Both functional suppression and refractive structural differences can significantly disrupt visual development (70). These findings underscore the importance of addressing binocular function in amblyopia treatment rather than focusing solely on the recovery of monocular visual acuity.

### 3.5 Strabismus

Strabismus is an ocular misalignment disorder characterized by non-parallel visual axes of the two eyes, resulting in the inability to fixate on the same target simultaneously. Studies have demonstrated a nasotemporal asymmetry in interocular suppression in strabismus: exotropia often exhibits more profound suppression in the nasal hemifield, while esotropia shows more profound suppression in the temporal hemifield. Moreover, stereopsis is negatively correlated with the degree of nasotemporal suppression asymmetry. Even after surgical or refractive correction restores ocular alignment, individuals with a history of strabismus frequently exhibit persistent interocular suppression, which continues to impair binocular visual function. Thus, the evaluation of binocular vision in strabismus should focus on both the magnitude and the pattern of interocular suppression (71). In contrast, Chung et al. investigated the relationship between ocular dominance and the anatomical structure of the lateral rectus muscle in intermittent exotropia (IXT) from a structural perspective. Except for a slightly narrower tendon width of the non-dominant eye compared to the non-dominant group (9.2 ± 0.7 mm vs. 9.4 ± 0.5 mm), no significant differences were observed between the two groups in all other parameters. This finding suggests that ocular dominance in IXT may be more closely associated with sensory or functional asymmetry rather than anatomical disparities in the lateral rectus muscle (72).

### 3.6 Age-related macular degeneration

Age-related macular degeneration (AMD), the leading cause of irreversible VFD in elderly populations, arises from complex gene–environment interactions. Binocular system medicine conceptualizes AMD as a systemic degenerative disorder affecting bilateral macular function, wherein interocular symmetry or asymmetry provides a critical window into disease mechanisms and therapeutic prognoses. OCTA-based evaluation of 1,310 AMD patients revealed that 54% demonstrated symmetric bilateral involvement, with significant interocular symmetry variations across AMD subtypes: the poorest symmetry was observed in drusen, exudative AMD (eAMD), and mixed disease, whereas geographic atrophy (GA) exhibited relatively higher bilateral concordance. In eyes with bilateral drusen, drusen area and volume measurements demonstrated moderate interocular symmetry (73). The study hypothesizes that symmetry in early AMD stages, such as drusen, may be driven predominantly by genetic factors. At the same time, asymmetry in advanced pathologies, such as GA and eAMD, likely reflects asymmetric environmental exposures during disease progression. Declining interocular symmetry with AMD progression underscores the clinical imperative to characterize bilateral asymmetry patterns for optimizing diagnostic frameworks and personalizing therapeutic strategies. In binocular system medicine practice, the routine assessment of AMD lesion symmetry status, which is evaluated through OCT parameters, lesion typology, and spatial distribution, constitutes a cornerstone for monitoring disease stability, predicting progression risk, and guiding individualized intervention timing (73).

In AMD therapeutics, intravitreal anti-vascular endothelial growth factor (VEGF) agents such as ranibizumab have become cornerstone interventions. A 7-year longitudinal study demonstrated that sustained anti-VEGF therapy delays visual decline and macular atrophy (MA) progression in neovascular AMD: study eyes receiving monthly ranibizumab for 2 years maintained superior visual outcomes compared to contralateral eyes. At 7-year follow-up, study eyes exhibited significantly smaller MA areas (2.8 ± 2.2 mm^2^ vs. 5.8 ± 2.5 mm^2^ in contralateral eyes), particularly in baseline neovascular AMD cases. Multivariate regression confirmed MA severity as an independent predictor of final visual acuity, with greater MA burden correlating with poorer vision in contralateral eyes. This study demonstrates that aggressive anti-VEGF therapy for exudative AMD eyes confers benefits beyond the treated eye: its effect in delaying MA progression indirectly suggests potential preservation of functional reserve within the entire binocular visual system (67).

### 3.7 Diabetic retinopathy

Diabetic retinopathy (DR), a microvascular complication of diabetes, necessitates early screening, detection, and therapeutic intervention to improve prognoses and prevent irreversible vision loss. Binocular system medicine conceptualizes DR as a localized manifestation of systemic metabolic dysregulation within the retinal microvasculature. The core pathophysiological feature, characterized by interocular microvascular network asymmetry, represents a critical determinant for understanding disease heterogeneity, progression dynamics, and personalized intervention strategies. Studies demonstrate progressive interocular asymmetry in vascular length density (VLD) and perfusion density (PD) across retinal layers among DR patients, with spatial heterogeneity amplifying linearly across disease stages: in SCP, interocular VLD disparity increases by 0.20 and PD by 0.004 per DR stage progression. In DCP, VLD increased by 0.33 and PD by 0.009 per stage (Table 1) (70). The superior quadrants exhibit the lowest VLD and PD values, while the inferior quadrant SCP VLD escalates by 0.41 per DR stage advancement (74). Notably, systematic interocular microstructural disparities are observed: left eyes demonstrate significantly reduced RBV density, lower fractal dimension, diminished circularity index, and greater FAZ structural disruption in the SCP, though FAZA remains comparable. As the disease progresses, absolute interocular FAZ parameter differences and asymmetry indices escalate with DR severity, reflecting widening pathophysiological divergence (75). Anatomic asymmetry may correlate with hemodynamic factors: differential flow patterns between left and right carotid systems, coupled with the left CCA’s aortic arch origin, may elevate left internal carotid pressure. This hemodynamic stress propagates via the ophthalmic artery, predisposing the left eye to microvascular compromise and bilateral asymmetry.

Meanwhile, male patients exhibit significantly higher interocular DCP VLD/PD asymmetry compared to the female ones. Sustained hyperglycemia (HbA1c > 6.5%) exacerbates FAZ circularity reduction (76). Consequently, precise quantification of interocular VLD/PD disparities, FAZ parameter asymmetry, and identification of left-eye microvascular network vulnerability enables early localization of the initiation site and severity gradient of retinal microvascular system imbalance. This binocular system medicine-guided approach facilitates early-stage risk stratification even when monocular manifestations remain subclinical. Therefore, in studies utilizing OCTA to analyze microvascular alterations in DR, systematic assessment of interocular asymmetry characteristics not only aids in identifying the laterality of initial pathological manifestations but also establishes a crucial empirical foundation for developing personalized risk stratification models. This approach demonstrates substantial clinical significance for enabling precision interventions in disease management.

### 3.8 Central serous chorioretinopathy

Central serous chorioretinopathy (CSC), a chorioretinal disorder characterized by RPE dysfunction and choroidal hyperpermeability, manifests as serous detachment of the neurosensory retina with or without concomitant pigment epithelial detachment (PED) (77). Notably, left eyes with CSC demonstrate a higher incidence of leakage points localized to the peripapillary area and closer proximity to the optic disc edge compared to right eyes. Such eyes frequently exhibit chronic CSC features, including more prolonged duration of the disease, a wider area of PED alteration, increased prevalence of outer retinal atrophy, and persistent subretinal fluid, despite relatively stable visual acuity. This anatomical-clinical correlation suggests that peripapillary choroidal vascular structural anomalies may contribute to chronicization in left-eye CSC through localized hydrodynamic alterations (78).

Therefore, within the clinical practice of binocular system medicine, identifying the leakage site location in CSC patients carries significant prognostic value as an early-warning biomarker. For patients exhibiting juxtapapillary leakage in the left eye, a prioritization protocol should be implemented, emphasizing system-stabilizing therapeutics targeting choroidal hyperpermeability and RPE dysfunction. This finding necessitates extended monitoring intervals to detect chronicity indicators. The therapeutic objective under the binocular system medicine framework is maximal containment of lateralized system imbalance to prevent cumulative irreversible structural damage.

### 3.9 Photoreceptor disorders

Achromatopsia (ACHM), an inherited retinal disorder manifesting at birth or during infancy, is clinically characterized by severe visual impairment, pendular nystagmus, photophobia, and complete color vision loss. OCT reveals variable structural disruptions in the foveal ellipsoid zone (EZ), with EZ integrity grading correlating strongly with functional visual deficits. Within the binocular system medicine framework, ACHM represents a congenital state of bilateral photoreceptor system functional loss driven by specific genetic defects. Studies demonstrate robust interocular symmetry in macular peak foveal cone density, intercell distance (ICD), and the coefficient of variation of ICD, outer nuclear layer thickness, and BCVA among ACHM patients (79, 80). While aging shows weak correlations with progressive foveal cone density reduction and EZ degeneration, the preserved anatomical-functional symmetry between eyes not only provides biomarkers for disease monitoring but also suggests comparable therapeutic potential in bilateral eyes. This symmetry enables the strategic utilization of contralateral eyes as internal controls to enhance statistical power in clinical trials (79).

Retinitis pigmentosa (RP), a hereditary retinal dystrophy marked by progressive degeneration of photoreceptors and RPE, exhibits distinctive hyperautofluorescent (hyperAF) rings with well-demarcated borders on short-wavelength fundus autofluorescence imaging. Binocular system medicine conceptualizes RP as a progressive bilateral photoreceptor-RPE system degeneration driven by genetic heterogeneity, wherein symmetry variations in disease patterns provide critical insights for deciphering genotype–phenotype correlations and guiding clinical management. Investigations reveal significant interocular asymmetry in hyperAF ring morphology across RP subtypes: Autosomal dominant RP (adRP) demonstrates the highest prevalence of asymmetric hyperAF patterns (23%), exceeding rates observed in autosomal recessive and X-linked RP (81). Notably, X-linked RP carrier female patients exhibit greater interocular disparities in BCVA, visual field radius, and EZ width compared to male patients, which could be likely attributable to skewed X-chromosome inactivation patterns in individual eyes (82). The interocular asymmetry signature of hyperAF rings efficiently triages candidates for targeted genetic testing. By integrating asymmetry patterns with inheritance types, binocular system medicine delivers refined individualized prognostic stratification and directs differential binocular surveillance strategies. Patients presenting with asymmetric hyperAF rings warrant prioritized targeted sequencing of adRP-associated pathogenic variants, complemented by cascade genetic screening of first-degree relatives. This approach holds critical value for genetic counseling and familial disease management.

### 3.10 Systemic diseases

Multiple systemic sclerosis (MS), a chronic inflammatory disorder of the central nervous system, is driven by dysregulation of the adaptive immune system, leading to inflammatory demyelination and progressive tissue damage (83). Within the binocular system medicine paradigm, MS represents a quintessential multifocal demyelinating disorder of the central nervous system. Its impact on the optic nerve and retina frequently manifests characteristic interocular asymmetry, offering a unique window into disease activity and neuroaxonal damage. Ocular investigations reveal no significant interocular differences in macular or peripapillary choroidal parameters among MS patients, with mean inter-eye differences of −3.53 μm in central macular choroidal thickness and 0.11% in CVI (84). This evidence indicates that choroidal parameters lack sensitivity as biomarkers of MS-associated system imbalance in binocular system medicine evaluation. Approximately 20% of MS cases present with acute optic neuritis (ON) as the inaugural manifestation (85). Recent studies have identified critical diagnostic metrics for MS-associated ON: inter-eye percentage difference (IEPD) and inter-eye absolute difference (IEAD). These parameters demonstrate superior discriminative value in MS diagnosis, with IEPD and IEAD values (both 0.71) in macular GC-IPL significantly exceeding those of macular RNFL and GCC. Notably, IEPD exhibits enhanced diagnostic stability in differentiating unilateral vs. bilateral MS-related ON compared to conventional parameters (86). In relapsing–remitting MS, ON-affected eyes exhibit accelerated multifocal visual evoked potential amplitude reduction rates (1.3% ± 0.7% faster than contralateral eyes) and twofold greater thinning velocities in the temporal RNFL sector, correlating positively with optic nerve demyelination severity (83). Recent research identifies alterations in the thickness of the internal retinal layer (IRL) as a valuable biomarker for the early diagnosis of MS. The IRL comprises the RNFL, GC-IPL, and the inner plexiform layer IPL. These structures constitute an unmyelinated extension of the central nervous system and maintain direct anatomical continuity with the brain. In MS, demyelinating lesions induce trans-synaptic degeneration, affecting retinal ganglion cells and their axons, ultimately leading to structural damage within the IRL. This pathological involvement is most pronounced within the central region of the neurodegenerative pathologies. MS patients exhibit significantly reduced average IRL thickness, reflecting the loss of ganglion cells and axons. Furthermore, greater interocular asymmetry in IRL thickness correlates with more severe functional deterioration. Conversely, the magnitude of asymmetry in the IRL constitutes a robust systemic indicator under the binocular system medicine framework, reflecting the global central nervous system disease burden and predicting functional outcomes. This asymmetry provides novel insights into spatially selective neurodegeneration patterns in demyelinating diseases. Patients with significant interocular IRL asymmetry demonstrate accelerated functional deterioration, providing novel insights into pathologically relevant retinal anatomy in demyelinating disorders (87).

Carotid-cavernous fistulas (CCF) represent pathological vascular shunts characterized by direct or indirect diversion of arterial blood from the carotid circulation into the cavernous sinus, resulting in elevated venous pressure within the sinus that subsequently induces increased IOP. A clinical study involving 60 patients (64 affected eyes) demonstrated that 64.06% of ipsilateral eyes exhibited elevated IOP, glaucoma, or suspected glaucoma, with secondary ocular hypertension (40.63%) primarily attributable to increased episcleral venous pressure. The mean IOP was significantly higher in affected eyes than in contralateral eyes (22.95 ± 7.1 vs. 15.11 ± 2.99 mm Hg). Notably, even in affected eyes with nominally normal IOP (10–21 mm Hg), a persistent interocular difference was observed (16.59 ± 3.06 vs. 13.67 ± 1.89 mm Hg), yielding a mean differential of 2.92 ± 2.29 mm Hg (Table 1). This asymmetry was more pronounced in indirect-type CCF cases, where affected eyes demonstrated significantly higher IOP compared to direct-type CCF (24.5 ± 6.96 vs. 20.1 ± 6.82 mm Hg). Given that such interocular pressure discrepancies frequently evade detection during routine clinical examinations, the study underscores the critical importance of serial IOP monitoring to mitigate risks of irreversible visual impairment (88).

Cognitive frailty (CF), operationally defined as the co-occurrence of physical frailty and mild cognitive impairment, is associated with elevated risks of dementia and mortality. As the retina constitutes an anatomical extension of the central nervous system, its structural alterations may mirror neurodegenerative processes. Furthermore, binocular system medicine establishes a pioneering pathway for investigating the neurobiological basis of CF: retinal neurostructural symmetry/asymmetry may serve as a proxy for interhemispheric connectivity and functional equilibrium in the brain. A cross-sectional study of 222 neurologically impaired patients without preexisting ophthalmic conditions demonstrated that a mean interocular GC-IPL thickness disparity exceeding 17 μm correlated positively with memory-associated CF and cognitive impairment. In contrast, RNFL interocular differences (mean: 5.2 μm) showed no significant associations with CF or cognitive decline. Physical frailty parameters exhibited no significant association with retinal asymmetry metrics. These findings suggest that GC-IPL asymmetry may reflect early-stage neurodegenerative cascades, like amyloid deposition and glial dysfunction, which serve as a potential biomarker for CF (89).

## 4 Conclusion and future directions

In ophthalmic research and clinical practice, binocular symmetry and asymmetry metrics have emerged as critical analytical tools for elucidating disease pathogenesis and evaluating biological behavior. These quantitative interocular disparity parameters provide novel diagnostic dimensions that transcend conventional monocular assessment paradigms, enabling enhanced early disease detection.

Operational guidance gaps persist for implementing specific biomarkers, such as the >8 μm GCC asymmetry threshold in glaucoma, where conventional screening relying on optic disc RNFL analysis encounters multiple limitations when incorporating binocular GCC assessment: prolonged examination time, increased patient costs. To optimize clinical utility, a tiered protocol is recommended: initial cost-effective RNFL screening for high-risk cohorts (e.g., ocular hypertension, diabetes), reserving binocular GCC asymmetry analysis only for cases with borderline monocular parameters. This strategy should be augmented by artificial intelligence (AI)-enabled clinical decision support systems that automatically flag threshold-exceeding asymmetries, integrate real-time annotations with electronic medical records, and reduce manual interpretation burden. Consequently, mandating standardized quantitative interocular asymmetry outputs across all imaging platforms remains essential for establishing consistent diagnostic benchmarks.

Technological innovations in OCT, particularly its ultrahigh-resolution capabilities, currently permit micron-scale visualization of retinal vascular networks. Emerging multimodal imaging integration platforms hold promise for automating binocular symmetry parameter extraction and enabling dynamic modeling, thereby advancing ophthalmology from traditional “diseased-eye-centric” paradigms toward comprehensive “binocular systemic evaluation” frameworks. Priority research directions should focus on the following: (1) establishing population-specific reference intervals for physiological interocular variations across age strata and ethnic groups through multinational collaborative studies and (2) refining analytical architectures to operationalize interocular asymmetry metrics into closed-loop systems integrating data acquisition, algorithmic interpretation, and clinical decision support. This evolving “binocular systems medicine” paradigm not only optimizes ophthalmic diagnostic-therapeutic pathways but may also catalyze interdisciplinary innovation in the early detection of systemic disorders through ocular biomarkers.
